# Prevention Admission into Nursing homes (PAN): study protocol for an explorative, prospective longitudinal pilot study

**DOI:** 10.1186/s12877-022-02885-z

**Published:** 2022-03-19

**Authors:** Andrea L. Koppitz, Susanne Suter-Riederer, Gabriela Bieri-Brünig, Heike Geschwinder, Anita Keller Senn, Frank Spichiger, Thomas Volken

**Affiliations:** 1 School of Health Sciences, Research&Development, University of Applied Science and Arts Western Switzerland HES-SO, Rue des Arsenaux 16a, 1700 Fribourg, Switzerland; 2 apnurse, Bahnhofstrasse 32a, 7310 Bad Ragaz, Switzerland; 3 Department of Nursing Homes of the City of Zurich (PZZ), Walchestrasse 31, Post Box 3251, 8021 Zurich, Switzerland; 4 Department of Endocrinology and Diabetology, Cantonal Hospital Winterthur, Brauerstrasse 15, 8400 Winterthur, Switzerland; 5 Institute of Health Science, Research&Development, Zurich University of Applied Sciences ZHAW, Katharina-Sulzer-Platz 9, 8400 Winterthur, Switzerland

**Keywords:** Caregiver, Frailty, Prevention, Quality of life, Geriatrics, Transition, Rehabilitation, Transitional care, Older people

## Abstract

**Background:**

In Switzerland, there is a lack of adequate rehabilitation services, and effective coordination, that take into account the multifactorial health risks of older people. The literature shows that the hospitalisation rate in rehabilitation facilities has increased in recent years and that a gender bias exists. Additionally, there is little or no evidence available on the effect that a post-acute care programme might have over an extended period on functioning, quality of life and the informal network of older people. Therefore, the aim of this trial is to evaluate the sustainability of post-acute care within three nursing homes in Zurich, Canton of Zurich, Switzerland.

**Methods:**

The Prevention Admission into Nursing homes (PAN) study is a explorative, prospective, longitudinal pilot trial based on a convenience sample of three long-term care facilities in the Swiss Canton of Zurich. The proposed pilot study will examine the effects of a post-acute care programme on people aged ≥65 years with a post-acute care potential ≥ three admitted to any of the three post-acute care units (*n* = 260). Older people of all sexes admitted to one of the post-acute care units and likely to be discharged to home within 8 weeks will be eligible for participation in the study. The primary endpoint is functionality based on the Barthel Index. The secondary endpoints are independency based on delirium, cognition, mobility, falling concerns, frailty, weight/height/body mass index, post-acute care capability, quality of life, and lastly, the informal network. As part of process evaluation, a qualitative evaluation will be conducted based on constructive grounded theory to specifically analyse how the experience of informal caregivers (*n* = 30) can contribute to a successful daily life 6 months after discharge.

**Discussion:**

We expect to observe improved functional status and independence after the post-acute care programme. The qualitative evaluation conducted with caregivers will complement our description of the transition of older people towards living at home.

**Trial registration:**

This study is registered in the German Clinical Trials Register under DRKS00016647 (registered on 23.05.2019).

## Background

The World Health Organization recommends strengthening the availability of rehabilitation services in health systems [1]. It is generally accepted that rehabilitation should improve the functions and adaptability of those affected through the coordinated use of medical, social, occupational, technical and pedagogical interventions [2, 3].

Various terms for rehabilitation can be found in the literature: post-acute care (PAC), rehabilitative transitional care, geriatric rehabilitation, slow stream rehabilitation and acute transitional care. This study focuses on PAC. PAC takes into account that low-intensity therapies can be used to individually prepare older people for their return home, specifically those who are unable to return home immediately after hospital discharge [4]. Therefore, PAC can occur in many settings, such as inpatient rehabilitation facilities, long-term care facilities or home care [5]. Since the introduction of Diagnosis Related Groups, it has been observed that acute hospitals are increasingly referring older people who need follow-up treatment to rehabilitation facilities, in addition to homes and outpatient facilities [6]. Conducting a comprehensive geriatric assessment is standard practice in PAC facilities for determining a treatment plan, which increases the probability that people will not be institutionalised [7–9]. Those directly affected want their care to focus on a well-functioning social network and improve their mobility [10]. However, the extent to which PAC positively affects patient outcomes over an extended period has not been extensively investigated [8].

Although improved functional status does not guarantee a return home, it is a central outcome of rehabilitation and subsequent treatments [11]. A decline in functional capacity can lead to undesirable events, such as hospitalisation. Monitoring functional status can help detect changes in the health status of chronically ill people at an early stage [12], and the functional status of older people is an essential indicator for rehospitalisation [13–16]. It has also been shown that older people with reduced functional status and cognitive abilities often need long-term care [15, 17–19]. Hence, maintaining functional status is a central influencing factor of the ability to live independently at home or with the assistance of a home-care organisation [12, 16, 20, 21]. Functionality in rehabilitation care is predominantly measured using the Barthel Index (BI). However, although the BI is widely used in rehabilitation care, it is not described within the PAC context [22].

PAC is becoming increasingly important in international health care settings [23–25]. PAC and rehabilitation services for older people after hospital stays in Switzerland were therefore implemented in 2011 and are governed by cantonal policies [26]. Some cantons authorise nursing homes to deliver PAC, while other cantons opt for PAC delivery by hospitals or home-care providers [27]. The Canton of Zurich has opted to provide PAC in nursing homes and does so successfully [22]. However, equitable access and rehospitalisation issues remain [28]. Moreover, unlike in rehabilitation clinics and hospitals, quality improvement and control for PAC in nursing homes is not centralised [29].

The hospitalisation rate in rehabilitation facilities in Switzerland has increased in recent years and currently stands at 10.1 per 1000 inhabitants [30]. In 2017, the most frequent reasons for admission of older people to rehabilitation facilities were injuries, musculoskeletal diseases and cardiovascular diseases [31]. Older people referred to inpatient rehabilitation have more illnesses that require more intensive rehabilitation [32, 33], while the average length of stay has decreased in recent years [30]. Older people undergoing rehabilitation have a primary diagnosis linked with an average of six secondary diagnoses [31]. This indicates a high co-morbidity rate among older Swiss people in rehabilitation.

Furthermore, in Switzerland, there is a lack of adequate rehabilitation services, and effective coordination, that take into account the multifactorial health risks of older people [34, 35]. Problems associated with the discharge of older people from Swiss hospitals may be related to the study results of Koné and colleagues [34]. They found that discharge planning in Swiss hospitals is not standardised. Also, hospitals refer women less frequently to rehabilitation institutions than men [34]. It is unclear why women are less often rehabilitated than men of the same age with similar conditions.

In summary, based on this multifaceted background, there is a lack of evidence as to a) to what extent a PAC programme positively affects patient outcomes, specifically the functional status, and b) how sustainable these effects are over an extended period. This knowledge is pivotal in informing future care pathways for this co-morbid population and enhancing adequate rehabilitation services with an effective PAC programme.

### Aims and hypothesis

The aim of this study is to evaluate the sustainability of PAC using functionality as the primary endpoint, measured using the BI. We hypothesise that the PAC programme improves a patient’s functional status (measured using the BI) to a level that allows them to return home after completing PAC in the nursing home and maintain a moderate increase in BI for at least 6 months after discharge (odds ratio = 1.5). Based on the study’s exploratory nature, we assume that moderate improvements are associated with a substantial gain in independence.

## Methods

The SPICE framework is used to describe the study components [36]. The version adapted for this study contains the following elements: S = study design, setting, sample size, P = participants, I = intervention/issue of interest, E = Endpoints, including their specific analyses. Comparison (C) has been omitted, as this is not a clinical trial.

### Study design and setting

The Prevention Admission into Nursing homes (PAN) study is a three-site, multicentre, exploratory, prospective, longitudinal pilot study in Swiss nursing homes without a control group. All centres (*n* = 3) are located in Zurich and the study will examine older people (*n* = 260) enrolled in PAC treatment. Additionally, a qualitative process evaluation will assess relatives’ perspectives (*n* = 30).

This study will be led by Zurich University of Applied Science’s health department and the department of Nursing Homes of the City of Zurich (PZZ) and will last approximately 24 months.

### Sample size

The sample size calculation was based on the primary endpoint, the BI, for which we model the cumulative probability that the i-th BI Y_it_ will fall into the j-th category or below:

1 $$logit\left(P\left({Y}_{it}\le j\right)\right)={\theta}_j-{\delta}_1\left(t{1}_{it}\right)-{\delta}_2\left(t{2}_{it}\right)-u\left({patient}_i\right)$$

i corresponds to the index for observations at time t, j represents the index for categories, {*θ*} is the cut-points, *δ*1 corresponds to the change in BI between PAC entry (baseline) and PAC exit, and δ2 is the change in BI between PAC entry and follow-up measurement at home after 6 months. Patient effects (random effects) are designed as independent and identically normally distributed:

2 $$u\left({patient}_i\right)\sim N\left(0,{\sigma}_u^2\right)$$

Of vital importance for the evaluation of the sustainability of PAC is parameter δ2, for which the following hypothesis will be tested:

3 $${H}_0:{\delta}_2\le \kern0.5em 0.4,\kern1.5em {H}_1:{\delta}_2>0.4$$

**Fig. 1:**
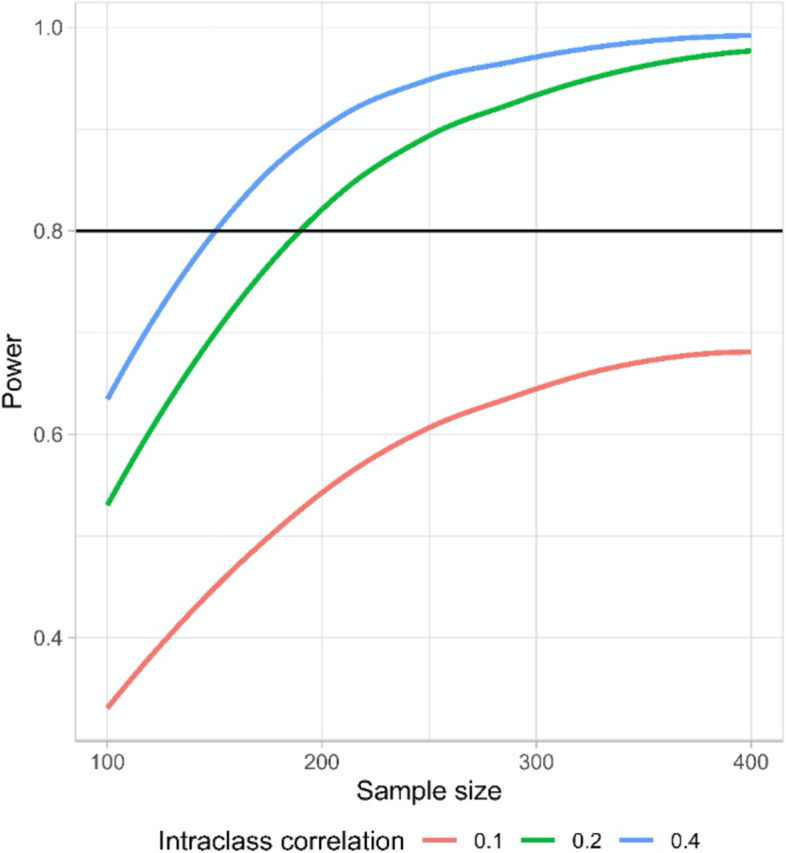
Power and sample size according to different intra-class correlations

The expected improvement in BI under the alternative hypothesis is >0.4 logits, which corresponds to an odds ratio (OR) of about 1.5. The required sample size to detect an effect under the alternative hypothesis was determined by simulation. We assumed a significance level of α = 0.05 (two-sided test) and a target power of 0.8 (β = 0.2). The obtained power is based on 500 iterative analogue model estimates and was performed for different levels of patient-specific autocorrelation (intra-class correlation [ICC] = 0.1, 0.2, 0.4). As is usual with mixed-effects models, the sample size decreases with increasing ICC (see Fig. 1).

Since no empirical values are available for the ICC from similar studies with comparable endpoints, we assumed a relatively conservative ICC of 0.2 to determine the sample size. Our assumption resulted in a calculation of 200 older people being needed to achieve the targeted power. Taking into account the dropout rate, which we estimate to be 30% due to the advanced age of the study population, the study requires a sample size of *n* = 260.

The coloured curves show three different estimates of obtained power based on our simulations. Each curve assumed another level of patient-specific autocorrelation. Our assumed target power of 0.8 is highlighted with a horizontal line.

### Participants

The study population will consist of older people enrolled in a PAC treatment programme and their informal caregiver network.

1. Older people

**Fig. 2:**
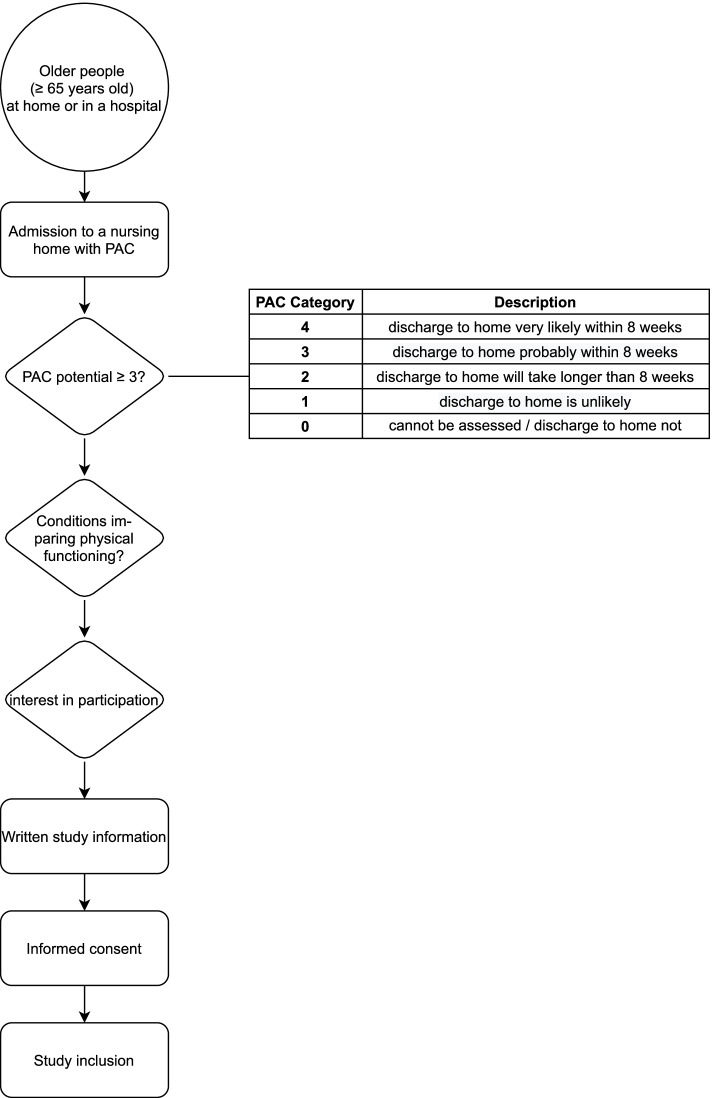
Recruitment process for post-acute care admission

The following inclusion criteria will be applied to the older people: must be ≥65 years old; admitted to one of the nursing homes and participating in the running PAC programme; assessed with a PAC potential ≥3 (see Fig. 2) and conditions impairing physical functioning; able to communicate in German; and must have provided informed consent.

2. Informal caregiver network

The following inclusion criteria will be applied to the informal caregiver network (i.e., primary relatives in charge or trusted persons): must have the capacity and the capability to provide informed consent; be able to communicate in German; and be of legal age.

For a description of the recruitment, refer to Fig. 2. The department of Nursing Homes of the City of Zurich (PZZ), namely the third author, will be in charge and administrate the study participants’ enrolment.

### Intervention

Typically, patients are selected for geriatric rehabilitation during their acute hospital stay (i.e., usual rehabilitation care). PAC provides a differentiated geriatric assessment and estimates the PAC potential for those affected by musculoskeletal diseases, injuries and circulatory diseases. PAC provides rehabilitation for older people who leave an acute hospital and are not sufficiently independent to resume living at home or can no longer cope at home. The aim is to help them reach the point at which they can reintegrate into their homes. Interprofessional teams conduct PAC programmes in nursing homes (see Fig. 2). Unfortunately, no uniform international definition of PAC exists [5]. Wang and colleagues (2019) proposed a clinical perspective with five goals: 1) the potential to live independently, 2) a comprehensive assessment, 3) enhancing patients independent living, 4) program duration 6 weeks, and 5) individual assessment mechanisms [5]. PAC treatment and plans with individual goals are developed according to the comprehensive assessment of cognition, nutritional status, physical function and mobility and basic activities of daily living [22]. Nursing, medical and therapeutic interventions and treatments support those affected in regaining the greatest possible independence. PAC gives older people more time than in a traditional rehabilitation setting to take on as much as possible by themselves and thus strengthen their self-care abilities.

Treatments with a rehabilitative approach are characterised by close interprofessional cooperation focusing on functional activities, education and psycho-social support [37]. Physicians are primarily responsible for PAC assessment, drug treatment, follow-up and evaluation. Therapeutic specialists, such as physiotherapists and occupational and speech therapists, are tasked with improving functioning, and nurses transfer and integrate (re)gained functions into the daily lives of older people. For example, the handgrip strength regained through targeted physiotherapy is transferred to everyday life through targeted nursing interventions. Patients are given sufficient time and individual support to select, prepare and eat their bread rolls for breakfast independently instead of simply feeding them. Overall, providing advice, particularly on mobility and self-care, to patients and their caregivers is an essential nursing intervention in PAC [37]. Social service providers carefully plan the return home and subsequent outpatient care. Furthermore, they initiate necessary measures in collaboration with the primary relatives.

A PAC programme lasts an average of 8 weeks and a maximum of 10 weeks based on the PAC potential assessment. If, after this time span, a return home is not possible, even with appropriate support, suitable alternatives are explored together with the affected person. In cases where a return home is likely after an extended stay, the older person is transferred to a specialised nursing ward to attain adequate independence and functioning.

### Endpoints

The following outcomes will guide the evaluation of the PAC programme.

1. Functional status as the primary endpoint

Functional status will be measured with the BI [38] at PAC entry (T0), PAC exit (T1) and 6 months after PAC discharge to the home environment (T3). The BI is a ten-item scale with either 0, 5, 10 or 15 points allocated for each item (maximum total score of 100 points = maximum functional status). The BI is a validated and common instrument used to assess functionality in rehabilitation care [38, 39]. For use with older and multi-diagnosed people, there might be imprecision when testing takes place by different observers [40]. To the best of our knowledge, little is known about the sensitivity to changes in the BI [41]. A clinically meaningful change is to be determined by the patients themselves. Recommended BI cut-off points to determine overall independence are problematic. Overall, a five-point difference can have completely different meanings for individual daily life independence. Collin and colleagues [39, 41] recommended that a 20-point change be considered as clinically significant. For the individual use of BI, other endpoints must also be considered.

All items will be collected through direct observation by study personnel or interviews with those affected, their relatives or the nursing staff.

2. Independence as the secondary endpoint

Independence will be measured via the informal network and the following nine criteria: delirium, cognition, mobility, falling concerns, frailty, body mass index (BMI), PAC capability and quality of life. The details of each criterion are as follows:

- Delirium: The assessment of delirium will be performed with the Delirium Observational Scale (DOS) at PAC entry (T0) [42]. The instrument has shown a high internal consistency with a reported Cronbach’s alpha = 0.96 and is considered valid and reliable for the early detection of delirium in older people [37, 38]. Delirium per shift will be evaluated using 13 items and four levels (never, sometimes, always or do not know). The occurrence of more than three criteria indicates a positive delirium screening and will trigger additional assessment.
- Cognition: The degree of cognitive performance will be measured using the Mini-Mental State Examination (MMSE) at admission (T0) and at home (T3) [43]. The reliability of the MMSE has been reported with a reported Cronbach’s alpha = 0.91 [44]. The sum of the MMSE spans from zero to 30 points, with 30 being the optimal result. Cognitive impairment is diagnosed at an MMSE sum of less than 24 points [43, 45, 46].
- Mobility: Mobility will be measured using the Short Physical Performance Battery (SPPB) at PAC entry and 6 months after release from the PAC to the home environment (T0, T3) [47]. SPPB has proven reliable with a Cronbach’s alpha = 0.87 [48]. The SPPB includes balance, a four-metre walking test and the stand-up test. A score of 10–12 points represents normal mobility, a score of 7–9 points indicates an increased risk of gait disorder and a score of less than 6 points indicates a very high risk of increasing gait disorder and loss of independence [47].
- Falling concerns: Concerns regarding falls will be measured with the German version of the Falls Efficacy Scale-International (FES-I) at PAC entry and 6 months after release from the PAC to the home environment (T0, T3). The German version of the FES-I has a high internal consistency (Cronbach’s α = 0.96) and retest-reliability (*r* = 0.96) [49]. The FES-I questionnaire consists of 16 items in which concerns about different activities and cases are raised. The answers are graded as follows: one = no concerns; two = some concerns; three = moderate concerns; and four = very great concerns. A minimum score of 16 indicates no concerns about falling, and a maximum score of 64 indicates severe concern about falling [49, 50].
- Frailty: Frailty will be assessed using the frailty screening questionnaire [51, 52] at PAC entry and 6 months after release from PAC to the home environment (T0, T3). The questionnaire has been shown to be a valid and reliable instrument with a test-retest reliability total score of *r* = 0.937 [53]. The frailty screening questionnaire examines five factors (weight loss, fatigue, walking speed, physical weakness and activity level). The assessment is “met” or “not met”, with zero factors met indicating robustness, one to two factors met indicating a pre-frail status and less than three factors met indicating frailty [51, 52].
- Weight/height/BMI: Weight, height and BMI measures will be used as co-variates of frailty [8, 54]. All three elements will be measured at entry and 6 months after release from PAC to the home environment (T0, T3) [8].
- PAC capability: PAC capability assessment will be conducted using the Resident Assessment Instrument for Nursing Homes (RAI_NH) to determine the degree of care dependency at PAC entry (T0) and at PAC discharge (T1). The “Minimum Data Set” category of the Swiss RAI_NH is a needs assessment tool [55]. The RAI_NH care dependency score ranges from tier 1 (lowest) to 12 (highest) [55]. All items will be collected through direct observation or interviews with those affected, their relatives or the nursing staff.
- Quality of life: Quality of life will be measured as a subjective screening of health status on a numeric rating scale ranging from “excellent (10)” to “poor (0)” as an influence on functionality. It will be measured at entry, discharge and 6 months after release from the PAC to the home environment (T0, T1 and T3).
- Informal network: The frequency of use of formal and informal caregiver networks to secure the home environment will be measured by interviewing the primary relative in charge. The frequency of informal network use will be measured 6 months after discharge from PAC to the home environment (T3).

Data from the study participants will be routinely collected four times (see Table 1). Socio-demographic data of the participants will also be collected, particularly to analyse sex-specific aspects.

**Table 1:** Outcome Measurements during the Intervention Schedule

Timepoint	T0	T1	T2	T3
	PAC admission	PAC discharge	3 months after discharge	6 months after discharge
**Primary Outcome**				
**· Barthel-Index**	+	+		+
**Secondary Outcomes**				
**· Delirium (DOS)**	+			
**· Cognition (MMSE)**	+			+
**· Mobility (SPPB)**	+			+
**· Falling concerns (FES-I)**	+			+
**· Frailty**	+			+
**· Weight, height, BMI**	+			+
**· PAC capability**	+	+		
**· Quality of life**	+	+		+
**· Informal network**				+

Ninety days after PAC discharge, telephone contact will be established with the older person to ask whether they want to continue participating in the study and to verify their address.

As part of the process evaluation, the primary relatives (*n* = 30) will be individually interviewed 6 months after PAC discharge using constructive grounded theory to identify impacts of the PAC programme on their quality of life during their daily routines [56]. These interviews will be conducted by trained PZZ staff members not involved in the PAC programme.

The timepoints are labelled T0 to T3. The plus symbol (“+”) indicates the timepoints at which each instrument will be applied. At T2, no outcomes will be evaluated, but the participants’ residence and continued participation will be verified.

### Statistical analysis

Data analysis will be performed using R-Project software version 3.5.2. The socio-demographic data will be analysed descriptively (frequencies, central tendencies, standard deviations). For the primary endpoint, the BI, we will estimate unadjusted cumulative odds using the basic model presented in the sample size section to detect BI changes between baseline and follow-up measures. The model will further be adjusted for age, sex, and secondary endpoints mobility and frailty, which have been identified as driving factors leading to institutionalisation in a nursing home. Both unadjusted and adjusted OR with corresponding 95% confidence intervals will be reported. Similarly, unadjusted and adjusted generalised linear mixed-effects models of the appropriate family and link function will be used to estimate the parameters of the secondary endpoints. Statistical significance will be established at *p* < 0.05.

### Analysis of interviews

Process evaluation analysis of the informal caregiver networks will apply constructive grounded theory [56], run through the Atlas.ti® software Version 8.4.4. Within constructive grounded theory, data analysis and data collection take place simultaneously, which is why data analysis will begin with the first interviews. Sample size depends on theoretical saturation. This leading criterion for the grounded theory method can vary [56]. In this case, we assume that we will interview up to 30 relatives. Using comparative strategies (so-called “constant comparisons”), text passages, events, strategies and persons will be compared to identify content similarities and differences. Further analysis will be conducted by employing two analytical steps: initial and focused coding. A data segment will be coded row by row during initial coding to develop categories. In the next step (focused coding), the properties and connections of the codes will be developed so that an explanatory model of daily routines after the PAC programme is created in an inductive process. The process will be documented using memos to ensure the credibility and quality of the analysis [56].

## Discussion

The complexity of older people’s treatment requirements and the lack of information about the sustainability of PAC in Switzerland highlight the urgent need for an evaluation of PAC. Current data do not permit any further differentiation of geriatric rehabilitation programmes and, more specifically, of PAC treatment programmes for people with injuries, musculoskeletal diseases and circulatory diseases [31–33]. Older people are often multimorbid. In Swiss university hospitals, 79% of patients (median age: 68 years old) are multimorbid and have seven types of illnesses (median), four of which are chronic (median): chronic heart disease, chronic kidney disease, solid malignancy and substance-related disorders. Each of these four types of illness comprises two to eight comorbidities. The most common are neurological diseases, heart/kidney diseases, malignancy, miscellaneous diseases and psychiatric diseases [57]. Therefore, this study aims to evaluate functional status using the BI as the primary endpoint. In addition, quality of life and the informal network (relatives) will be assessed as secondary endpoints.

Nevertheless, there are some points to discuss. First, a programme of 6 weeks for multimorbid patients is often too short to gain independence and depends on the severity of the impairments. Second, improved functions are not always clinically significant. There may be a difference between performing in a laboratory setting and coping with daily life. An effective program always takes place in interaction with the environment [58]. Third, for people with neurological diseases, for example, dealing emotionally with role changes due to physical limitations or reduced social participation is essential for living as independently as possible at home [59, 60].

PAC programmes include repeated assessments, individual treatment and therapy plans (e.g. physiotherapy, occupational therapy and nursing). The therapies take place individually and in groups. In assessment interviews, older people and their family members are informed about their status, treatment progress and plan. Discharge planning is also important. Older people in PAC receive information and documents on continuing care, medications, therapies and aids as needed. Their general practitioners are also informed so that further medical care is ensured.

If the results of this study show that PAC programmes delivered in nursing homes improve the participants’ functional status to the point that they can return home, then the tested programmes could be considered an effective way to delay the elderly from permanently residing in a nursing home. Furthermore, having evidence of a successful PAC programme will offer specialised nursing homes the opportunity to differentiate their services and transform their image from the “last station in life”. Having access to a comprehensive geriatric assessment tool will also enable PAC staff to develop treatment plans, which decreases the probability that older people will be institutionalised [7, 9].

According to the British Medical Research Framework for complex interventions [61], this study is categorised as a pilot study to determine whether the PAC programme can improve older people’s functional status, as measured using the BI. Moreover, the repeated measures study design allows the assessment of functionality over several months and ensures that the intervention’s short-term and long-term effects can be adequately captured and differentiated [62].

In sum, we will determine the sustainability of the PAC programme from the perspectives of the elderly and their relatives over 6 months. We expect that the PAC programme will improve the functional status of older people and reduce the burden on their relatives.

## Data Availability

The datasets produced as part of the study are available on request from the corresponding author.

## Abbreviations

BI: Barthel Index
BMI: Body Mass Index
DOS: Delirium Observation Scale
FES-I: Falls Efficacy Scale-International
MMSE: Mini-Mental State Examination
PAN: Prevention Admission into Nursing homes
PAC: Post-acute Care
PI: Principal Investigator
SPPB: Short Physical Performance Battery
RAI_NH: Resident Assessment Instrument for Nursing Homes
